# Commencing Nutrient Supplements before Full Enteral Feed Volume Achievement Is Beneficial for Moderately Preterm to Late Preterm Low Birth Weight Babies: A Prospective, Observational Study

**DOI:** 10.3390/nu10101340

**Published:** 2018-09-20

**Authors:** Wei Qi Fan, Amy Gan, Olivia Crane

**Affiliations:** 1 Department of Paediatrics, The Northern Hospital, 185 Cooper Street, Epping, VIC 3076, Australia; 2 Faculty of Medicine, Dentistry and Health Sciences, The University of Melbourne, Grattan Street, Melbourne, VIC 3010, Australia; amyggan@gmail.com (A.G.); olivia.k.crane@gmail.com (O.C.)

**Keywords:** moderately preterm, late preterm, human milk fortification, LBW, blood urea, EBM, preterm formula, SCN, SCBU

## Abstract

The aim of this study was to observe after following a routine change in the feeding protocol whether the earlier introduction of nutrient supplements improved nutritional outcomes in moderately preterm to late preterm low birth weight (LBW) babies. In this prospective observational study, LBW babies between 31 and 39 weeks’ gestation admitted to a Special Care Nursery were assigned to two groups (F80, *n* = 45, F160, *n* = 42) upon commencing nutrient supplement at total fluid intake achievement of 80 or 160 mL/kg/day. Outcomes included weight, protein intake, biochemical markers, feeding intolerance, and length of stay (LOS). F80 nutrient supplements commenced before F160 (2.8 vs. 6.7 days, *p* < 0.0001) and lasted longer (15.2 vs. 12.2 days, *p* < 0.03). Weight gain velocity and LOS were similar. F80 mean protein intake during the first 10 days was higher (3.38 vs. 2.74 g/kg/day, *p* < 0.0001). There were fewer infants with protein intake <3 g/kg/day in the F80 group (8% vs. 65%, *p* < 0001). F80 babies regained birthweight almost two days earlier (7.5 vs. 9.4 days, *p* < 0.01). Weight gain Z-scores revealed an attenuation of the trend towards lower weight percentiles in the F80 group. Feeding intolerance was decreased for F80 (24.4% vs. 47.6%, *p* < 0.03). There were no adverse outcomes. Earlier nutrient supplementation for LBW babies lifts mean protein intake to above 3 g/kg/day and reduces both the duration of post-birth weight loss and incidence of feeding intolerance.

## 1. Introduction

In the Special Care Nursery (SCN), the feeding and nutrition of the moderate preterm (32 to 34 weeks’ gestation) to late preterm (34 to 36 weeks’ gestation) low birth weight (LBW) babies are key objectives. While more research has been directed toward the nutritional needs of extremely and very low birth infants (ELBW and VLBW) in the setting of the neonatal intensive care unit (NICU), there has been relatively less research on the nutritional needs of moderate to late preterm infants.

Although late preterm infants may be regarded by parents, caregivers, and health care professionals as being developmentally mature and at low risk of morbidity, this is often not the case [1]. Compared to full term infants, late preterm infants with poor feeding are seven times more likely to have delayed home discharge [2] and almost twice as likely to be readmitted for dehydration [3]. Although stabilization of suck and suck-swallow rhythms generally mature before 36 weeks’ gestation, coordination of respiration and swallow starts to improve later [4], which clearly has implications for the development of mature feeding. The establishment of breastfeeding in the late preterm infant compared to the full-term infant is often problematic due to immaturity and difficulties with latching, sucking, and swallowing [5]. Assessing feeding adequacy requires careful attention via a cue-based approach with an expressed breast milk (EBM) fortifier and preterm formula of potential benefit especially in those babies who are growth retarded and with gestation ages around 34 to 35 weeks [6].

In our SCN, babies are admitted from around 31 weeks and can have birth weights as low as 1200 g. Up until late 2014, we had a protocol of not commencing fortification of expressed breast milk (EBM) or preterm formula until full enteral feeds of 160 mL/kg/day had been achieved. However, there has been a shift towards commencing fortifier or preterm formula earlier in SCN and NICU feeding protocols in Australia with commencement at enteral feed achievement of 80 mL/kg/day or 100 mL/kg/day being more common [7]. As studies have shown that earlier nutrient supplementation is well tolerated [8], we made a clinical decision to also introduce earlier supplementation commencing in October 2014.

We were interested in assessing whether this earlier introduction of protein and mineral supplements would have any measurable outcome for our moderately preterm and late preterm nursery population. Consequently, we decided before the full introduction of earlier supplements to run a prospective observational study.

## 2. Materials and Methods

### 2.1. Overview

This study followed moderately preterm and late preterm LBW neonates during SCN admission between October 2014 and May 2016 at The Northern Hospital (TNH) in Epping, which is an outer suburb of Melbourne, Australia.

The catchment area for TNH is a lower-socioeconomic, multicultural, multi-ethnic community with a high rate of primary care health concerns such as obesity and admissions to our SCN reflect heterogeneous social and demographic factors. This presents a challenge when carrying out basic observational research in determining a representative sample reflecting community demographics. In this study, we used a simple random sampling technique that has been demonstrated to reliably reflect the community population [9] in observational studies. It should be emphasized that this is an observational study. No placebo or active comparator was involved [10]. Both protocols were identical in terms of the material provision of nutrient supplement and were run in parallel. To determine an appropriate sample size, we assumed that the weight gain velocity and LOS may be similiar, but earlier nutrient supplements may improve the regaining of post birth weight. Power calculation indicated a sample size of 35 in each group to detect an improvement of 2 days in the time to regain birthweight (alpha = 0.05, with a power of 80%).

### 2.2. Participation

Parents were informed of the study and gave written consent. Participants were allocated to respective groups by the joining sequence: i.e., infants achieving total fluid intake of 80 mL/kg/day (F80, *n* = 45) and infants achieving total fluid of 160 mL/kg/day (F160, *n* = 42). Inclusion criteria were: birthweight less than 2500 g, gestational age 31 weeks to term, and admission before tolerating a total enteral fluid intake of less than 80 mL/kg/day. Infants with gastrointestinal malformations or recognized chromosomal abnormalities were excluded. Babies were followed until SCN discharge. Ethics approval was provided through the Northern Health Human Research Ethics Committee in 2014.

### 2.3. Outcomes and Feeding Intolerance

Primary outcomes were considered to be: protein intake, days to regain birthweight, the rate of feeding intolerance and the incidence of Necrotizing Enterocolitis (NEC). NEC is identified in our nursery by systemic, intestinal, and radiological signs with disease severity based on Bell Staging criteria [11].

Secondary outcomes included weight gain velocity, length of stay (LOS), neonatal complications, and presumed or diagnosed sepsis. Infants were reviewed daily by medical staff for feeding intolerance, NEC, and sepsis. Feeding intolerance was defined by frequent large volume vomits with abdominal distension. Sepsis was presumed if there were systemic symptoms such as respiratory distress, fever, lethargy, and increased inflammatory markers such as C-reactive protein levels and full blood examination. Small for gestational age (SGA) was defined as weight being below the 10th percentile for gestational age using Fenton growth charts throughout the study.

### 2.4. Feeding Protocol

Healthy babies commenced feeding from day 1 at an enteral volume of 60 mL/kg and increased by 20 mL/kg daily as tolerated.

Human milk fortifier (FM85, Nestle, Vevey, Switzerland) was introduced into expressed breast milk (EBM) or preterm formula was commenced if EBM was not available (Pre-nan, Nestle, Vevey, Switzerland) or S26 low birth weight Formula (Aspen Nutritionals Pty Ltd., Clayville, South Africa) at respective total fluid intakes. FM85 was given at the recommended concentration of 1 g per 20 mL of EBM, which provided 1 g protein per 100 mL. Preterm formula provided a protein content of 2.9 g/100 mL. Fortification or preterm formula was given to all babies up to a weight of 2000 g. Thereafter, cessation was at the discretion of the neonatal consultant and, based on such issues as appropriate weight gaining, extent of feeding intolerance and SGA.

### 2.5. Data Collection and Calculation Assumptions

Weight was measured every third day by using scales accurate to 1 g. Weight measurements were single only and not replicated and averaged. Z-scores were calculated for birth weight, lowest weight post birth, weight at 10 days, and discharge weight using the 2013 Fenton growth charts [12]. Head circumference and length were measured by on duty nursing staff as routinely required, but this data was excluded from analysis as unreliable due to inconsistency.

Standard biochemical and hematological tests were performed at day 7 and 21 of life and included hemoglobin, reticulocyte count, alkaline phosphatase (ALP), blood urea, and creatinine. Since this was an observational study, from time to time, routine requests for blood tests were either not initiated by error or an insufficient sample was collected, which resulted in laboratory test data not being available for all study participants. Techniques for measuring milk protein levels analytically were not available to us, so protein content was calculated based on two factors: EBM and gestation age using published data [9]. For example, EBM at a gestational age of 33 weeks provided protein content of 2.2 g/100 mL and EBM at a gestational age of 34 weeks 2.1 g/100 mL [13]. Each day the total milk requirement of a baby was calculated. The volume of consumed breastfed breast milk was estimated to be the same as the unconsumed volume of EBM or preterm formula left over from the daily calculated input. During the study period, the predominant method of feeding was EBM via the feeding tube.

Since we were not using analytical techniques to measure milk protein levels and were relying on published data, we thought it prudent to use another methodology to help validate the protein calculations. We did this through routine urea tests done on study participants as a normal practice in our SCN. In the presence of normal renal function, blood urea reflects protein intake [14]. Normal renal function in the newborn is achieved by 21 days [15]. However, day 7 blood urea levels can be corrected. We used the method of Moro et al. [16]: day 7 blood urea levels were corrected by multiplying the observed day 7 blood urea level by the averaged cohort day 21 creatinine level modified by a ratio to the observed day 7 creatinine level. Averaged blood creatinine in our study at 21 days was 33 μmol/L. With regard to relating blood urea levels to protein intake levels, we used the findings of Polberger et al. [17]. They followed infants who had been born between 26 and 32 weeks but waited, on average, 19 days until they had achieved equivalent gestational ages between 32 to 36 weeks. The study period was 28 days. Protein enrichment was commenced at an enteral volume of 170 mL/kg/day. They found a high correlation between blood urea levels and protein intake. We have adopted their finding that, for protein intake less than 3 g/kg/day, blood urea levels were always less than 1.6 mmol/l. Even though their equivalent gestational ages of 32 to 36 were similar to our study, their study did not report the actual weight of infants during their study period.

Being enrolled for 10 days in the study was considered a minimum for data analysis.

### 2.6. Statistical Methods

Groups were compared using *t*-tests for continuous variables and chi-square or exact tests for categorical variables. A two-sided significance level was set at *p* < 0.05. Data was tested for normality by using skewness and kurtosis and found to be acceptable. NCSS statistical software (NCSS Statistical Software, Kaysville, UT, USA) was used.

## 3. Results

### 3.1. Study Population and Demographic Characteristics

A total of 115 babies were enrolled in the study. However, 28 babies were not included for data analysis since they did not complete the first consecutive 10 days of the study. The F80 group had 45 participants and the F160 group had 42 participants.

For the total cohort, the infant gestational age ranged from 31 to 39 weeks and birth weight ranged from 1380 g to 2496 g. Both groups were comparable in relation to infant birth weight, discharge weight, gestational age, sex, mode of delivery, intrauterine growth restriction, and the frequency of maternal gestational diabetes mellitus. See Table 1.

### 3.2. Anthropometric Findings

The F80 group regained birthweight almost two days earlier. Weight gain as measured by a change in the Z-score from birth showed that a significantly lower decrease in weight percentiles occurred for the F80 group. Otherwise, there was no significant difference in maximum weight loss following birth, weight gain velocity, or LOS. See Table 2.

### 3.3. Nutrition and Feeding Outcomes

**Figure 1:**
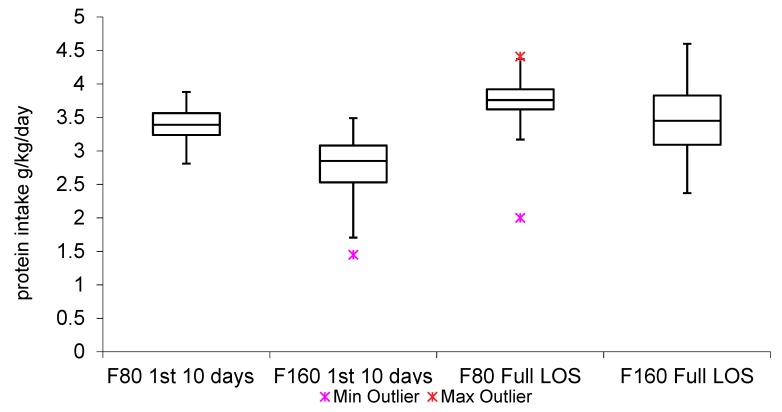
Box plot of protein intake for the F80 and F160 groups during the first 10 days of feeding and for the F80 and F160 groups over the full LOS. Daily protein intake for the first 10 days—Median (F80: 3.89, F160: 2.85). Daily protein intake for the first 10 days—IQR (F80: 0.33, F160: 0.55). Protein Intake daily per full LOS—Median (F80: 3.76, F160: 3.45). Protein Intake daily per full LOS—IQR (F80: 0.30; F160: 0.75), LOS, length of stay. 1st, first. IQR, inter quartile range.

See Table 3. All babies received some standard formula or preterm formula as a top up. Almost all babies received human milk (92% overall). 80.5% of babies were breast fed at some stage. Of those breastfed, there were six breast feeding episodes on average per LOS with 19% of babies having 10 or more breast feeds per LOS. A total of 70% of breast feeding episodes were of a volume less than 50 mL. F80 babies commenced nutritional supplements earlier and for a longer duration. Over the first 10 days, the F80 group received increased protein intake and there were significantly fewer babies failing to receive 3 g/kg/day. Additionally, the variance of the distribution of protein intake was significantly less over the first 10 days. This finding is explored further in Figure 1. Over the full LOS, this increase in protein intake was maintained. However, over the full LOS, there was no difference in the frequency of protein intake <3 g/kg/day. Figure 1 illustrates the protein intake for both F80 and F160 groups over the first 10 days and over the full LOS.

### 3.4. Blood Urea as a Marker of Protein Intake Adequacy

**Figure 2:**
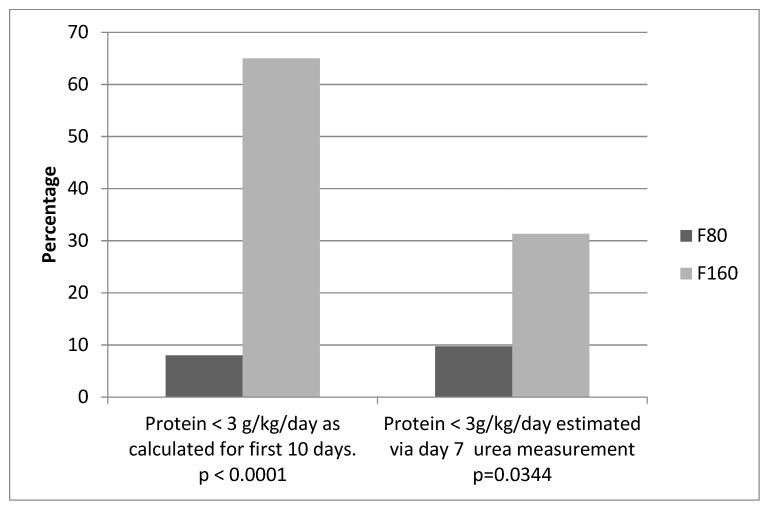
Frequency of infants with protein intake <3 g/kg/day calculated versus estimated via blood urea measurement. χ^2^ test for probability.

At seven days of life, there was no mean difference in blood urea levels between the two groups, but, when using a cut off level of 1.6 mmol/L, markedly fewer babies in the F80 group had estimated protein intake levels less than 3 g/kg/day (See Table 4 and Figure 2). 

### 3.5. Clinical Outcomes

Feeding intolerance in the F80 group was significantly less. There were no differences in the frequencies of presumed sepsis, hypoglycaemia, the phototherapy requirement, and the blood ALP levels. There was no occurrence of NEC in any of the study subjects. See Table 5.

## 4. Discussion

The simple expedient of commencing routine nutrient supplementation earlier resulted in a number of benefits. As thought possible, the earlier introduction of the nutrient supplement was associated with an almost two day earlier regaining of birthweight. Earlier supplementation was also associated with improved protein intake. This improvement was marked in the first 10 days, but also lasted the length of the SCN stay. During the first 10 days, the number of babies in the early supplementation group receiving less than 3 g/kg/day of protein was significantly decreased. Although growth as assessed by weight gain velocity was not different, Z-score analysis showed that earlier supplementation significantly mitigated the extent of post birth weight percentile reduction at both 10 days and at discharge. Surprisingly, an earlier nutrient supplement was associated with a 50% improvement in feeding intolerance.

A key component of both human milk fortifier and preterm formula is protein. However, compared to VLBW and ELBW babies, there has been relatively less research on the protein needs of the moderate and late preterm infant. Theoretically, protein intake should be important. Consider a theoretical example. A baby born at the 50th percentile at 34 weeks’ gestation has a birthweight of approximately 2000 g and has a projected normal term birthweight of approximately 3200 g [18]. Assuming two weeks to regain a healthy birthweight, a growth rate of around 15 g/kg/day has been calculated to parallel fetal growth rates culminating in 3000 g bodyweight at 40 weeks post menstrual age [19], which is a process requiring adequate protein intake. Additionally, protein intake also helps minimize weight loss due to energy requirements immediately after birth. Up until late gestation, while glucose metabolism is important, the fetus has a low ability to metabolize fat. Instead, oxidation of amino acids provides significant energy [20]. Consequently, the earlier provision of protein/amino acids soon after birth is important in maintaining energy and minimizing protein store degradation [20]. Even in babies born at term, the irreversible loss of protein during fasting has been estimated at 0.87 g/kg/day [21].

For moderately preterm infants, enteral protein intake necessary to achieve fetal rates of growth have been quoted at 3.6 g/kg/day for 1500 g to 1800 g infants and 3.4 g/kg/day for 1800 g to 2200 g infants [22]. Our study (Table 3) showed that, during the first 10 days, F80 mean protein intake was consistent with achieving such targets. Importantly, in our study, only 8% of the F80 group had calculated protein intake less than 3 g/kg/day compared with 65% of the F160 group. This finding was corroborated by the blood urea results, which also showed the F80 group to have a majority of protein intake above 3 g/kg/day at day 7 (see Table 4 and Figure 2).

Commencement of fortifier or preterm formula at an enteral volume of 80 mL/kg/day altered the dynamics of the distribution of protein intake (Figure 1). Babies in the F80 group experienced a much tighter distribution than those in the F160 group within the first 10 days and throughout the course of the hospital stay. This observation is supported by the highly significant difference in the variance of the two groups (Table 3). Stated another way, comparatively more babies in the F80 group received higher and consistent protein intake, which indicated an earlier and long-lasting toleration of protein. This contributed to the F80 group in regaining birthweight earlier (Table 2) and is a possible part explanation for the improved feeding intolerance (Table 5). With regard to the earlier regaining of birthweight in the F80 group, obvious factors that may have been an influence can be discounted. First, the mean birthweight of both groups was almost identical (2028 g vs. 2032 g). There was no evidence of transient tachypnoea of the newborn and no babies in the study received IV fluids. Supporting the changed dynamics of protein intake and the improvement in the rates of protein intake above 3 g/kg/day are the Z-score findings for weight, which showed a significant mitigation in the F80 group of the trend toward lower weight percentiles at both 10 days and at discharge.

Feeding intolerance is the inability to digest enteral feedings. It is associated with symptoms such as increased gastric residuals and abdominal distension. It often leads to the disruption of an infant’s feeding plan and presents a challenge to the clinician in providing adequate nutrition. Additionally, there is the potential that its occurrence may signal the onset of necrotizing enterocolitis NEC [23]. In this context, the almost 50% reduction in feeding intolerance observed in this study is an important finding. The reason for this beneficial reduction in feeding intolerance associated with earlier nutrient supplementation is not clear even though some recent studies’ findings allow speculation and provide a possible future research direction. Feeding intolerance relates to a disorganized premature intestinal tract and a multiplicity of factors such as swallowing reflex, lactase enzyme activity, an immature motility pattern, and the establishment of intestinal flora, which have a key role in gut barrier function and the immune response [24]. In relation to intestinal flora, it has been shown that late preterm infants have a significantly different microbiome than full term infants [25] and that there is a growing body of evidence that intestinal bacteria may have a causative role in NEC [26]. Micronutrients in nutritional supplements have a role to play in both physiological intestinal maturation and in the developing intestinal microbiota. For example, zinc has been shown to have trophic effects on intestinal mucosa, modulates intestinal permeability, and zinc deficiency has implications for NEC [27]. Additionally, animal studies such as in the neonatal piglet, have shown that dietary zinc level can have an inhibitory effect on some intestinal flora species and is capable of influencing the composition of the intestinal microbiota [28]. Could it be that the earlier introduction of micronutrients in our study occurred at a threshold point in the complex, multifactorial developing intestine, which promotes a level of intestinal stability that diminishes if introduction is delayed by a few days?

There was no statistical difference in LOS between the study groups (Table 2). This observation is unsurprising since LOS was determined by the requirement for discharge that weight achieved should be 2300 g or more and that post menstrual age should be at least 36 weeks. Despite the positive nutritionally-related benefits observed in the F80 group, the F80 group did not achieve full enteral feeds earlier than the F160 group. This result is also unsurprising since the feeding regime was identical for both groups and required 20 mL/kg/day grade up in milk volumes. Additionally, although the F160 group had a higher incidence of feeding intolerance, feeding tolerance mainly manifested after full enteral feeds were achieved.

This study showed that earlier nutrient supplementation boosted protein intake, reduced feeding intolerance, and was associated with an improvement in weight growth curves. The question then arises as to whether these short-term improvements are likely to have positive longer-term benefits. Brain development could be one such possibility. During the final six to eight weeks of gestation, brain size increases by more than a third. There is also a five-fold increase in white matter volume from 35 weeks’ gestation and significant development of white matter microstructural integrity [29,30]. The importance of dietary protein has been demonstrated in a study on preterm infants with white matter injury (WMI), which showed that a high energy and protein diet during the first year after birth resulted in a significant improvement in head growth, weight gain, and corticospinal tract axonal diameters [31]. Even though WMI such as periventricular leukomalacia more commonly occurs in very premature infants, it also occurs in the moderate or late preterm infant [29] and is linked to cognitive deficits later in life [32]. WMI is associated with inflammation and ischemia, which suggests that nutritional supplements may have a role in reducing systemic infections and associated inflammatory responses promoting a reduction in WMI and improved brain development [33].

The results of this study and discussion of possible future directions and implications highlights the need for many further studies into the nutritional requirements of moderate-preterm to late-preterm infants and medium to long term outcomes. While some studies have already examined longer term aspects in such preterm infants such as the prevalence of hypertension and diabetes [34,35], there is still much left to research. A proposal for one such study was recently announced [36], which aims to assess the effects of feeding strategies for moderate to late preterm infants on body composition, feed tolerance, and neurodevelopmental outcome.

The major limitation in this study was the use of published figures to estimate protein levels of expressed breast milk and the subtractive method of estimating the volume of breast feedings.

## 5. Conclusions

Commencing human milk fortifier or preterm formula at half enteral feed volumes (80 mL/kg/day) resulted in a number of benefits for the moderate to late preterm baby. Protein intake was lifted and consistently consumed both in the short term and for an infant’s LOS to levels that have been recognized as adequate for growth. Additionally, there was a reduction both in the time taken to regain birth weight, an improvement in growth curves, and, importantly, a reduction in feeding intolerance. There were no negative clinical outcomes.

## Figures and Tables

**Table 1:** Comparative demographics.

	F80 (*n* = 45)	F160 (*n* = 42)	*p* Value
Birth Weight (g) ± sd	2028 ± 235	2032 ± 297	0.95
Birth Weight Z-score ± sd	−0.737 ± 0.91	−0.578 ± 0.97	0.43
Birth Gestation (weeks) ± sd	34.4 ± 2.1	34.2 ± 1.6	0.74
Discharge Weight (g) ± sd	2312 ± 255	2300 ± 263	0.83
SGA	19 (42%)	11 (26%)	0.12
Males	26 (52%)	20 (48%)	0.34
Maternal GDM	9 (20%)	13 (31%)	0.24
Caesarean Birth	29 (64%)	18 (43%)	0.07

Mean and percentage data. Student’s *t*-test for birth weight & gestation. χ^2^ for all other categories. sd, standard deviation, SGA, *Small* for gestational age (defined as below 10th percentile for weight at gestational age), GDM, Gestational diabetes mellitus. Z-score: derived from Fenton growth charts for preterm infants.

**Table 2:** Anthropometric findings.

	F80 (*n* = 45)	F160 (*n* = 42)	*p* Value
Maximum Weight Loss (g) ± sd	108 ± 69	124 ± 59	0.95
Weight Loss as % Birth Weight	5.3%	6.1%	
Weight Loss Δ Z-score ± sd	−0.531 ± 0.28	−0.639 ± 0.25	0.08
Days to Regain Birth Weight ± sd	7.5 ± 3.2	9.4 ± 2.8	<0.01
Weight Gain Velocity (g/kg/day)	7.3 ± 5.5	7.0 ± 4.2	0.76
Weight Gain: Δ Z-Score at 10 Days ± sd	−0.661 ± 0.18	−0.762 ± 0.22	0.03
Weight Gain: Δ Z-Score at Discharge ± sd	−0.620 ± 0.27	−0.765 ± 0.23	0.02

Mean and percentage data. Student’s *t*-test for all categories. sd, standard deviation. Z-score: derived from Fenton growth charts for preterm infants. *n* = 38 for F80 and *n* = 37 for F160 Z-score data (*n* reduced due to missing data points). Δ Z-score: Change in Z-score from birth.

**Table 3:** Nutrition and feeding outcomes.

	F80	F160	*p* Value
TP Intake daily 1st 10 days (g/kg/day) ± sd	3.38 ± 0.26	2.74 ± 0.53	<0.0001 *
TP Intake daily 1st 10 days—Variance	0.07	0.28	<0.0001 **
TP Intake daily 1st 10 days <3 g/day	8%	65%	<0.0001 ***
TP Intake daily per LOS (g/kg/day) ± sd	3.69 ± 0.46	3.44 ± 0.52	0.0245 *
TP Intake daily per LOS—Variance	0.21	0.27	0.2280 **
TP Intake daily per LOS <3 g/day	11%	17%	0.6629 ***
Fortifier commencement day ± sd	2.8 ± 0.8	6.7 ± 1.8	<0.0001 *
Length of fortification (days) ± sd	15.2 ± 6.1	12.2 ± 6.5	0.0273 *
Number babies fed fortified EBM	42 (93%)	38 (91%)	0.9242 ***
F80 days to reach 160 mL/kg ± sd	6.8 ± 1.2	6.7 ± 1.8	0.8288 *

TP, total protein, mean values. Sd, standard deviation. TP Intake 1st 10 days—*n* = 38 for F80, *n* = 37 for F160. (*n* reduced due to missing data points). Fortifier Commencement day—day post birth at which fortifier or preterm formula was commenced. F80 day to reach 160 mL/kg—the number of days the F80 group took to reach 160 mL/kg milk intake. LOS, hospital length of stay—*n* = 45 for F80, *n* = 42 for F160. Number babies fed fortified EBM—these babies all had some standard or preterm formula top up. *n* = 45 for F80. *n* = 42 for F160. * Student’s *t*-test, unpaired data, mean values. ** F-test—two-sample for variances. *** χ^2^ test.

**Table 4:** Blood urea as a marker of protein intake adequacy.

	F80	F160	*p* Value
Urea 1st week (raw data) ± sd	4.1 ± 1.5	3.6 ± 1.9	0.2087 *
Urea 1st week (corrected) ± sd	3.6 ± 2.0	3.15 ± 2.0	0.3642 *
Urea (corrected) 1st week <1.6	9.7%	31.3%	0.0344 **

Urea, blood urea in mmol/L. sd, standard deviation. 1st week data: *n* = 33 for F80, *n* = 35 for F160. Urea (corrected) = Urea (raw data) × 33/blood creatinine (raw data). Urea levels <1.6 mmol/L correspond to enteral protein intake <3 g/kg/day. * Student’s *t*-test, unpaired data, mean values. ** χ^2^ test.

**Table 5:** Frequency of Clinical Outcomes.

	F80 (*n* = 45)	F160 (*n* = 42)	*p* Value
Feeding intolerance Jaundice requiring phototherapy	11 (24.4%) 12 (26.7%)	20 (47.6%) 18 (42.9%)	0.0276 * 0.1124 *
Presumed sepsis	17 (40.0%)	17 (40.5%)	0.9639 *
Hypoglycaemia	3 (6.7%)	4 (9.5%)	0.6244 *
NEC	0	0	1.0
ALP at 7 days ± sd	256.7 ± 86.9	229.4 ± 80.5	0.1659 **

NEC—necrotizing enterocolitis. ALP—alkaline phosphatase. sd = standard deviation. * χ^2^ test. ** Student’s *t*-test, unpaired data, mean values. Hypoglycemia defined as plasma glucose <2.6 mmol/L.
